# *Plasmodium malariae* structure and genetic diversity in sub-Saharan Africa determined from microsatellite variants and linked SNPs in orthologues of antimalarial resistance genes

**DOI:** 10.1038/s41598-022-26625-w

**Published:** 2022-12-19

**Authors:** Eniyou C. Oriero, Martha A. Demba, Mouhamadou F. Diop, Deus S. Ishengoma, Lucas N. Amenga-Etego, Anita Ghansah, Tobias Apinjoh, Soulama Issiaka, Abdoulaye Djimde, Umberto D’Alessandro, Martin Meremikwu, Alfred Amambua-Ngwa

**Affiliations:** 1 Medical Research Council Unit The Gambia at LSHTM, Fajara, Gambia; 2 National Institute for Medical Research, Dar es Salaam, United Republic of Tanzania; 3 University of Ghana, Legon, Accra, Ghana; 4 Noguchi Memorial Institute for Medical Research, Accra, Ghana; 5 University of Buea, Buea, Cameroon; 6 Centre National de Recherche et de Formation sur le Paludisme, Ouagadougou, Burkina Faso; 7 Centre National de Recherche Scientifique et Technologique, Ouagadougou, Burkina Faso; 8 University of Science, Techniques and Technology, Bamako, Mali; 9 University of Calabar Teaching Hospital, Calabar, Nigeria

**Keywords:** Genetics, Molecular biology, Diseases, Medical research

## Abstract

*Plasmodium malariae*, a neglected human malaria parasite, contributes up to 10% of malaria infections in sub-Saharan Africa (sSA). Though *P. malariae* infection is considered clinically benign, it presents mostly as coinfections with the dominant *P. falciparum*. Completion of its reference genome has paved the way to further understand its biology and interactions with the human host, including responses to antimalarial interventions. We characterized 75 *P. malariae* isolates from seven endemic countries in sSA using highly divergent microsatellites. The *P. malariae* infections were highly diverse and five subpopulations from three ancestries (independent of origin of isolates) were determined. Sequences of 11 orthologous antimalarial resistance genes, identified low frequency single nucleotide polymorphisms (SNPs), strong linkage disequilibrium between loci that may be due to antimalarial drug selection. At least three sub-populations were detectable from a subset of denoised SNP data from mostly the mitochondrial cytochrome *b* coding region. This evidence of diversity and selection calls for including *P. malariae* in malaria genomic surveillance towards improved tools and strategies for malaria elimination.

## Introduction

The genetic diversity and population structure of human pathogens, often derived from molecular genotyping, are useful for monitoring their local and regional transmission patterns and the effect of public health interventions. The population structure of major malaria parasite species are assumed to be driven by geographic isolation, human migration, malaria transmission intensity, and selective pressure by drugs and vector interventions^1^. An understanding of the distribution of malaria parasites, particularly with respect to candidate drug resistance markers in endemic areas, will further inform the design of malaria control and elimination intervention strategies. Such knowledge has often targeted the two main human malaria species, *Plasmodium falciparum* and *P. vivax*, while others such as *P. malariae* have been largely neglected. A limited number of studies on genetic diversity of *P. malariae* in sub-Saharan Africa reported high diversity of the parasite species in different endemic regions, but no population structure or significant differentiation was found^2,3^. These studies included mostly infections from sites not very far apart, such as was reported for two endemic villages in Malawi that are about 100 km apart^2^. With additional sources for isolates across sub-Saharan Africa and more neutral genetic markers, a broader view of the diversity of this species could be provided.

Targeted characterisation of gene loci has also been explored to describe *P. malariae* populations. Genetic variations in *P. malariae* antigenic proteins thrombospondin-related anonymous protein—TRAP, apical membrane antigen 1—AMA1, and 6-cysteine protein—P48/45 were reported in Asia (Thailand, Myanmar and Lao)^4^. These showed high mutational diversity in *P. malariae trap* and *ama1* compared to *p48/45,* with mostly non-synonymous mutations that suggest genetic variation in these genes is maintained by positive diversifying selection^4^. Similarly, new non-synonymous mutations were observed in polymorphic sites of the *dhfr* gene, leading to the description of six new candidate DHFR alleles associated with sulfadoxine-pyrimethamine (SP) resistance^5^. This has been further shown in recent *P. malariae* whole genome sequence analyses that reported four synonymous and seven non-synonymous single nucleotide polymorphisms (SNPs) in some orthologs of drug resistance genes^6^. All these studies suggest high diversity in *P. malariae* parasite species as well as frequent recombination and gene flow between populations. To further investigate this, we explored the genetic diversity and population structure of *P. malariae* across seven endemic countries in sub-Saharan Africa using highly polymorphic microsatellite loci and describe the SNPs in orthologous genes associated with antimalarial resistance in *P. falciparum*.

## Methods

### *P. malariae* samples

Archived blood samples at the Medical Research Council Unit The Gambia (MRCG) and those from collaborators within the Pathogen Diversity Network Africa (PDNA) were accessed following mutual consent and ethical permissions. The *P. malariae* infected samples analyzed were from seven sub-Saharan countries with varying malaria transmission settings and antimalarial drug use and resistance profiles (Table 1). Samples processed were either dried blood spot or already extracted DNA, except for those from Nigeria where leucocyte depleted red blood cell (RBC) were used. DNA was extracted using QIAamp DNA mini kit (QIAGEN, Germany) before confirmatory real-time PCR diagnosis of *P. malariae* infection was done as previously described^7^. The resulting 75 confirmed *P. malariae* infections (mostly co-infected with *P. falciparum* by qPCR analysis) were used for further genetic diversity analysis as shown in Table 1.

**Table 1:** Distribution of *P. malariae* samples from endemic countries.

S/N	Country	Region	Year of collection	Sample type	Transmission setting	Antimalarial drug used	No. of *P. malariae* samples
1	Nigeria	West Africa	2017	Leucocyte depleted RBCs	High	AL	18
2	Tanzania	East Africa	2009–2017	DBS	Moderate	AL	19
3	Burkina Faso	West Africa	2014–2015	DNA	High	ASAQ/DHAP/AP	17
4	Mali	West Africa	2017	DBS	High	AL	9
5	Guinea	West Africa	2014	DBS	High	AL	4
6	Cameroon	Central Africa	2015	DBS	High	ASAQ/AL	4
7	Ghana	West Africa	2017	DNA	Moderate	AL/ASAQ	3
8	Unknown	S.E Asia	Unknown	DBS	Unknown	Unknown	1

*AL* Arthemeter-Lumefantrine, *ASAQ* Artesunate-Amodiaquine, *DHAP* Dihydroartemisin piperaquine), *AP* Artesunate-Pyronaridine.

### Microsatellite amplification and fragment analysis

Multi-locus genotyping of *P. malariae* was carried out using five highly divergent microsatellites (Pm_02, Pm_09, Pm_11, Pm_34 and Pm_47) in a semi-nested PCR reaction as previously described^2^. The primary amplification contained 3 µL of *P. malariae* DNA in a 15 µL reaction, 1 × Thermopol buffer (New England Biolabs), MgCl_2_ at 1.5 mM, 0.6 U of Taq Polymerase (New England Biolabs), 0.8 mM of dNTPs and 0.05 μM each of forward and reverse primer. Same conditions applied for the semi-nested reaction using 2 μL of the primary amplification product in a 35 μL reaction with forward and reverse primer concentration at 0.1 μM. Cycling conditions for the primary amplification were—initial denaturation for 4 min at 94 °C followed by 35 cycles of denaturation for 30 s at 94 °C, annealing at 48 °C for 30 s, extension for 60 s at 68 °C and then a final extension for 2 min at 68 °C. The same cycling conditions were used for the semi-nested amplification except for annealing at 52 °C and a total of 20 cycles. Microsatellite PCR products were separated on a 3730 capillary sequencer (Applied Biosystems) following dilution (1:50–1:100) and post-amplification mixing of differently labelled and sized loci. Analysis of electropherograms was carried out using Geneious Prime Version 2022.0.1 (http://www.geneious.com) and alleles were scored automatically in comparison with size standard HD400 (Applied Biosystems).

### Targeted amplicon sequencing of antimalarial resistance genes

Eleven *P. malariae* orthologues of *P. falciparum* antimalarial resistance genes were amplified and sequenced (Table 2). Specific primers were designed for each of the genes and optimum conditions for amplification with Q5 Polymerase (New England Biolabs) are shown in Table 3. Amplified products were verified on 1% agarose gel and all amplicons were pooled per sample for deep sequencing library preparation using the TruSeq HT library prep kit. Libraries were cleaned with the Agencourt AMPure XP PCR purification kit (Beckman Coulter, Brea, CA, USA) according to the manufacturer’s protocol. Amplicon concentration and size were measured on a Qubit fluorometer (Invitrogen, Carlsbad, CA, USA) and Tapestation (Agilent). Paired-end sequencing was performed on the MiSeq (Illumina, San Diego, CA, USA) with 10 pM concentration of pooled amplicons, at the MRCG genomics platform, using the Illumina v2 reagent kit to produce 250 bp reads per end, according to manufacturer’s instructions.

**Table 2:** List of orthologous *P. malariae* antimalarial resistance genes sequenced.

S/N	Antimalarial resistance gene	Abbreviation	PlasmoDB ID
1	Amino acid transporter AAT1, putative	*Pmaat1*	PmUG01_11034100
2	AP-2 complex subunit mu, putative	*Pmap2mu*	PmUG01_14053100
3	Non-SERCA-type Ca^2+^-transporting P-ATPase, putative	*Pmatp4*	PmUG01_13021900
4	Calcium-transporting ATPase, putative	*Pmatp6*	PmUG01_02017400
5	Bifunctional dihydrofolate reductase-thymidylate synthase	*Pmdhfr*	PmUG01_05034700
6	Hydroxymethyldihydropterin pyrophosphokinase-dihydropteroate synthase, putative	*Pmdhps*	PmUG01_14045500
7	Kelch protein K13, putative	*Pmkelch13*	PmUG01_12021200
8	Multidrug resistance protein 1, putative	*Pmmdr1*	PmUG01_10021600
9	Sodium/hydrogen exchanger, putative	*Pmnhe*	PmUG01_14020100
10	Chloroquine resistance transporter, putative	*Pmcrt*	PmUG01_01020700
11	Cytochrome *b*, putative	*Pmcytb*	PmUG01_MIT001100

**Table 3:** Optimized conditions for generating the *P. malariae* drug resistance amplicons.

S/N	Primer	Sequence	Amplicon size (bp)	Primer (µM)	*MgCl_2_ (mM)	dNTP (mM)	Ext. time (s)	Tm (°C)
1	Pm_AAT1_F	AAATGGGTCAGTAGCCGCCTATG	1708	0.5	2.0	0.2	90	68
2	Pm_AAT1_R	ATCAGTTTGCGATTCATGTGTGCT						
3	Pm_CRT_F	AAAGTGACACACCTTATAGAGACC	729	0.5	2.0	0.2	90	66
4	Pm_CRT_R2	GCGAAGAACTGAAGCCCAAAA						
5	Pm_AP2mu_F	CCGTTTCGACAAGAAGTAATTC	1527	0.5	2.0	0.2	90	62
6	Pm_AP2mu_R	ACATACCACTGGAGGTAAACATAG						
7	Pm_ATP4_F	AACAAGAGAATCGTCTGAAAGG	3823	0.3	2.0	0.2	90	62
8	Pm_ATP4_R	AGCCCATGAAATGCCAAAGAGATA						
9	Pm_ATP6_F	TGACTGGGGAATCTTGTTCA	3699	0.5	3.0	0.7	90	62
10	Pm_ATP6_R	TCAATAATGATAACAGGAAAAGACCA						
11	Pm_CYTB_F	ACATGGTAGCACTAATCCTTTAGG	585	0.5	2.0	0.2	90	63
12	Pm_CYTB_R	CAGAAATATCGTCTTATCGTAGCC						
13	Pm_DHFR_F	TATGCCATCTGCGCTTGCT	1811	0.3	2.0	0.2	90	62
14	Pm_DHFR_R	TTATCATGGTGCACGTAATTTTG						
15	Pm_DHPS_F	ATACGAAACCGTCCCGGAGT	1897	0.5	2.0	0.2	90	68
16	Pm_DHPS_R	ACTGTACGAGGCAATGGCTAATCC						
17	Pm_Kelch13_F	CTGTCACGTATGATAGAGAATCC	2089	0.5	2.0	0.2	90	63
18	Pm_Kelch13_R	ATCAGCACAGAATGCCCAAATCTT						
19	Pm_MDR1_F	TATGTGCAACAATATCAGGAGG	4168	0.3	2.0	0.2	90	62
20	Pm_MDR1_R	ATACCATCCTGTTCTGCAAGTAGC						
21	Pm_NHE_F	TTTAGCAAACCTGGGCAGTTCTTG	4988	0.5	3.0	0.7	120	67
22	Pm_NHE_R	GTTAGCAATAGTCCATTGGCTGC						

*Q5 Polymerase buffer has 2.0 mM MgCl_2_.

### Data analysis

#### Microsatellite or simple sequence repeat (SSR) analysis

Binned microsatellite alleles were imported as genind objects into R statistical software (Version 4.1.13) and used for population genetic analysis. We initially defined populations as the countries from which the isolates were collected. Population-level genetic diversity was assessed with expected heterozygosity and number of alleles per locus (i.e., allelic richness). Values for expected heterozygosity range from 0 to 1 (0 indicating no diversity and 1 indicating all alleles are different). Diversity parameters such as observed and expected multilocus genotypes, Shannon–Wiener Index of multilocus genotype (MLG) diversity^8^, Stoddart and Taylor’s Index of MLG diversity^9^, Simpson’s Index^10^, Nei’s unbiased gene diversity^11^ and Evenness^12,13^ were calculated using the summary function “poppr” in R^14^. Pairwise Bruvo’s genetic distance was calculated with the microsatellite markers for all isolates using the command “bruvo.dist” in R and visualized as a hierarchical heat map. To determine population structure, the optimal number of genetic clusters was first determined by running k-means sequentially with increasing values of k and comparing different clustering solutions using Bayesian Information Criterion (BIC) in R software. In addition, the ‘find.clusters’ function in R’s adegenet package (version 2.0) was used to assign each individual into genetic clusters. Discriminant analysis of principal component (DAPC) was applied to describe clusters of genetically related individuals and displayed using scatter plots. DAPC transforms the data using principal component analysis (PCA), and then performs a discriminant analysis on the principal components retained using a cross-validation method. Ancestry was determined with the admixture model using STRUCTURE Version 2.3.4^15^ with individuals assigned to *K* populations based on their multilocus genotype. The estimated *K* likelihood value was set to 1–9 and STRUCTURE was used with 100,000 MCMC Reps over 10,000 Burn-in period. The most appropriate value of *K* was determined using calculated *ΔK* implemented in Structure Harvester Web v0.6.94^16^. Ancestry proportions were then visualized as bar plots.

#### Targeted candidate drug resistance gene sequencing and SNP analysis

**Figure 1:**
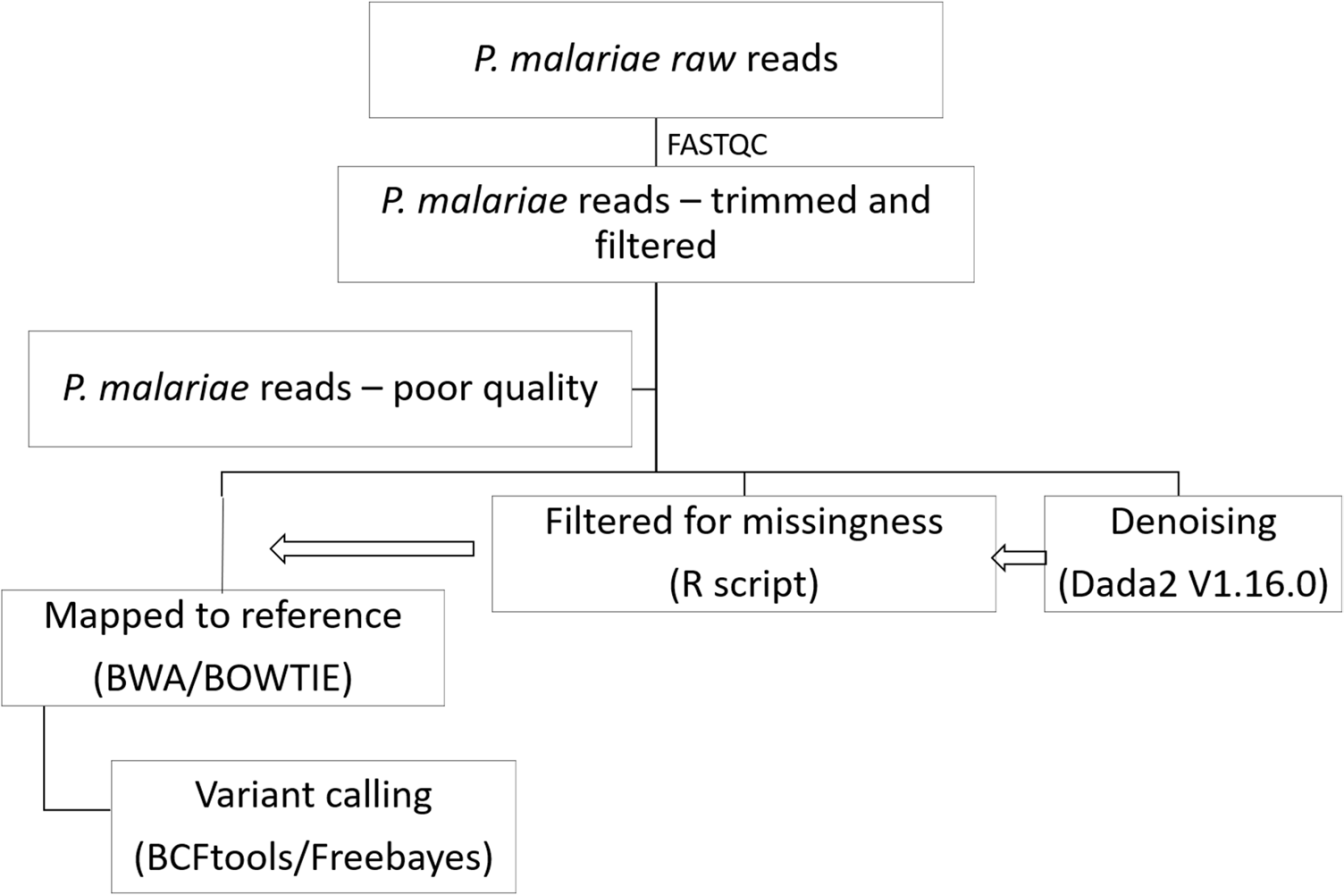
Process of amplicon sequence analysis.

The quality of Fastq files obtained was checked using FASTQC, then trimmed and filtered to remove poor quality segments and indices. The reads were processed as shown in Fig. 1—reads were either mapped directly to the reference of concatenated target genes obtained from PLASMODB OR filtered for missingness before mapping to the reference OR passed through a denoising pipeline (dada2 version 1.16.0) before filtration and reference mapping. Filtration for missingness was done in three steps (i) samples with greater than 80% missingness were removed from the original dataset (ii) loci with greater than 70% missingness from the dataset obtained from (filtration 1) were removed, and (iii) samples with greater than 40% missingness from the dataset obtained from (filtration 2) were removed (summarized in Supplementary Table 1).

Reads were mapped as merged or unmerged using both BWA and BOWTIE separately. Variant calling from each bam file was done using both BCFtools and freebayes. The resulting vcf files of variants were compared and concordant SNPs were identified for further analysis. To determine the structure of isolates from SNPs, DAPC was applied as described above and the first two coordinates visualised on a scatter plot. Pairwise population comparisons of the *P. malariae* orthologous drug resistance SNPs by Nei’s genetic distance were visualised as a hierarchical heat map. SNP-based genetic complexity within samples (complexity or multiplicity of infection) was assessed by estimating Wright’s inbreeding co-efficient (*Fws*). The *F*_*WS*_ metric (values range from 0 to 1) is a measure of within-host diversity that describes the relationship between the genetic diversity of an individual infection relative to the genetic diversity of the parasite population^17^. A low *F*_*WS*_ indicates high within-host diversity relative to the population. The extent of linkage disequilibrium (LD) between SNPs of the orthologous drug resistance targets was also determined and displayed using the LDheatmap R package.

## Results

### Genetic differentiation, population structure, ancestry and complexity of infection

All five microsatellite loci were highly polymorphic, with 8–12 alleles and a mean of 9.8 alleles per locus. Pm_09 was the most polymorphic microsatellite and Pm_11 was the least variable (Table 4). The expected heterozygosity (Nei’s genetic diversity) averaged across all loci was 0.72, ranging from 0.61 (Pm_11) to 0.85 (Pm_02). This measure provides an estimation of the probability that two randomly selected genotypes are different on a scale of 0 (no genotypes are different) to 1 (all genotypes are different). Mean genotypic evenness was 0.65; evenness is a measure of the distribution of genotype abundances, where populations dominated by a single genotype have values closer to zero and populations with equally abundant genotypes yield values close to one^18^.

**Table 4:** Summary statistics of microsatellite loci showing genotypic richness, diversity, and evenness by locus.

Locus	Allele	1-D	Hexp	Evenness
Pm_09	12	0.75	0.76	0.63
Pm_34	9	0.67	0.68	0.63
Pm_11	8	0.6	0.61	0.52
Pm_02	10	0.84	0.85	0.84
Pm_47	10	0.7	0.71	0.63
Mean	9.8	0.71	0.72	0.65

*1-D* Simpson’s diversity Index, *Hexp* Nei’s unbiased gene diversity.

At the country population level, only two pairs of MLGs were found in the isolates from Burkina Faso and Nigeria. Genotypic diversity and richness were high as indicated by high lambda or Simpson’s Index and expected heterozygosity—Nei’s unbiased gene diversity (Table 5). The least heterozygosity was observed for isolates from Cameroon (0.47), while the highest was for isolates from Ghana (0.80), with both including only 3 isolates. A total of 20 private alleles were observed across all populations with 50% of them seen in Tanzania. Genotypic evenness was high across populations (overall E.5 = 0.972). There was a wide variation in linkage disequilibrium between loci in populations as determined by the Index of Association (IA), ranging from 0 in Ghana to 1 for Cameroonian isolates.

**Table 5:** Summary statistics of microsatellite loci showing genotypic richness, diversity, and evenness by population.

Pop	N	MLG	eMLG	SE	PA	H	G	lambda	E.5	Hexp	IA
Burkina Faso	17	16	9.67	4.71e−01	3	2.75	15.2	0.934	0.969	0.746	1.28e−01
Cameroon	3	3	3.00	0.00e + 00	0	1.10	3.0	0.667	1.000	0.467	1.00e + 00
Ghana	3	3	3.00	0.00e + 00	2	1.10	3.0	0.667	1.000	0.800	−1.11e−16
Guinea	4	4	4.00	0.00e + 00	1	1.39	4.0	0.750	1.000	0.667	− 5.56e−01
Mali	9	9	9.00	0.00e + 00	2	2.20	9.0	0.889	1.000	0.733	5.16e−01
Nigeria	18	17	9.71	4.56e−01	2	2.81	16.2	0.938	0.970	0.703	2.83e−01
SE_Asia	1	1	1.00	0.00e + 00	0	0.00	1.0	0.000	NaN	NaN	NaN
Tanzania	19	19	10.00	2.51e−07	10	2.94	19.0	0.947	1.000	0.722	− 1.11e−01
Total	74	70	9.93	2.54e−01	20	4.23	66.8	0.985	0.972	0.722	1.01e−01

*N* number of individuals observed, *MLG* number of multilocus genotypes (MLG) observed, *eMLG* number of expected MLG at the smallest sample size ≥ 10 based on rarefaction, *SE* standard error based on eMLG, *PA* number of private alleles, *H* Shannon–Wiener Index of MLG diversity, *G* Stoddart and Taylor’s Index of MLG diversity, *lambda* Simpson’s Index, *E.5* Evenness, E5E5.

**Figure 2:**
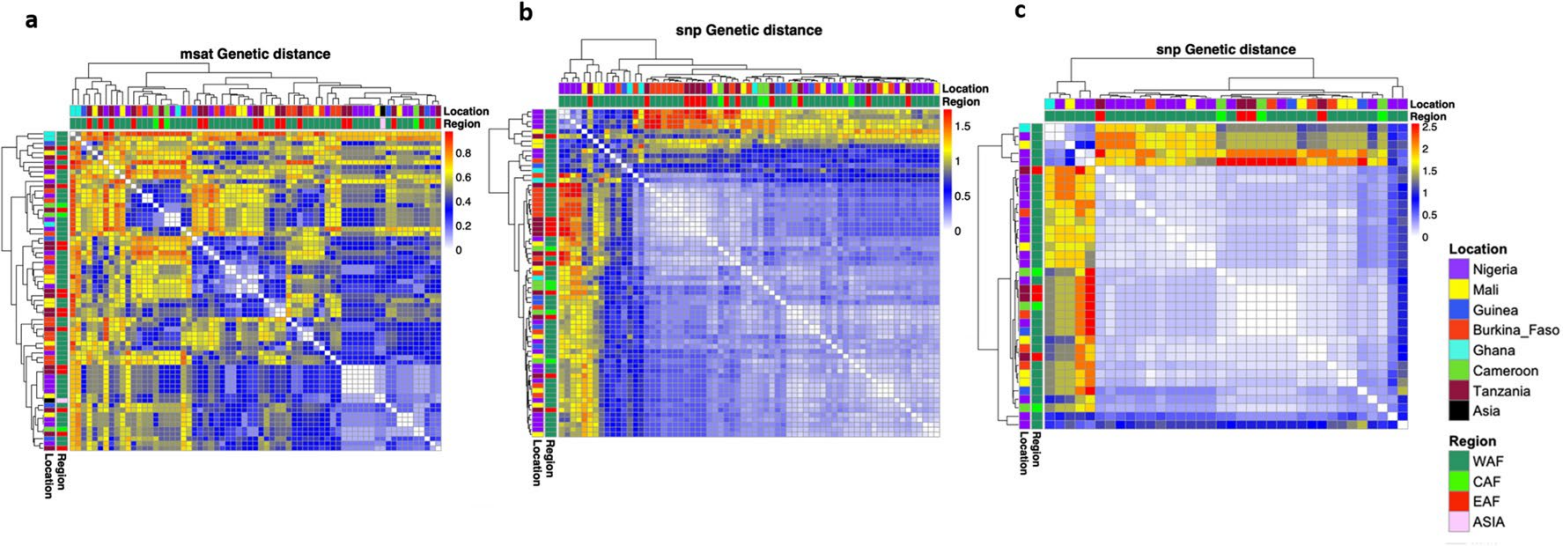
Clustering and heatmap of pairwise genetic distances computed using the pheatmap_1.0.12 package in R Version 4.1.13, (**a**) Bruvo’s distance from microsatellite (Msat) (**b**) Nei’s genetic distance between individual samples from SNPs in candidate drug resistance loci (unfiltered data) and (**c**) Nei’s genetic distance computed from SNPs after denoising and filtration of reads. Each sample’s origin by country or geographic region is shown as side bars at the branch ends of dendrograms.

The hierarchical heat map of genetic distances derived from microsatellite and SNP data indicated that the relationship among the *P. malariae* isolates was independent of their geographic origin (Fig. 2), with West, Central and East African isolates clustering together in most of the clades. Three genetic clusters were observed with the SNP data, which showed overall lower higher genetic distances due to the low frequencies of minor alleles and this was more evident with the filtered and denoised datasets (Fig. 2b,c). The distribution between the SNP and microsatellite distance differs, with wider distances between smaller number of isolates observed with the microsatellite data (Supplementary Fig. 1). This is expected as microsatellites are multi-allelic, likely neutral, and more likely to differ between pairs of isolates. Estimates of Wrights fixation index (*F*_*ST*_) of differentiation between populations was biased by small sample sizes but were low overall, and indication of poor differentiation between country populations due to intrapopulation genetic variation. The SNP data however showed relatively high genetic distance between Cameroon (which had the smallest number of sample) and Burkina Faso (*F*_ST_ = 0.437) (Supplementary Fig. 2).

**Figure 3:**
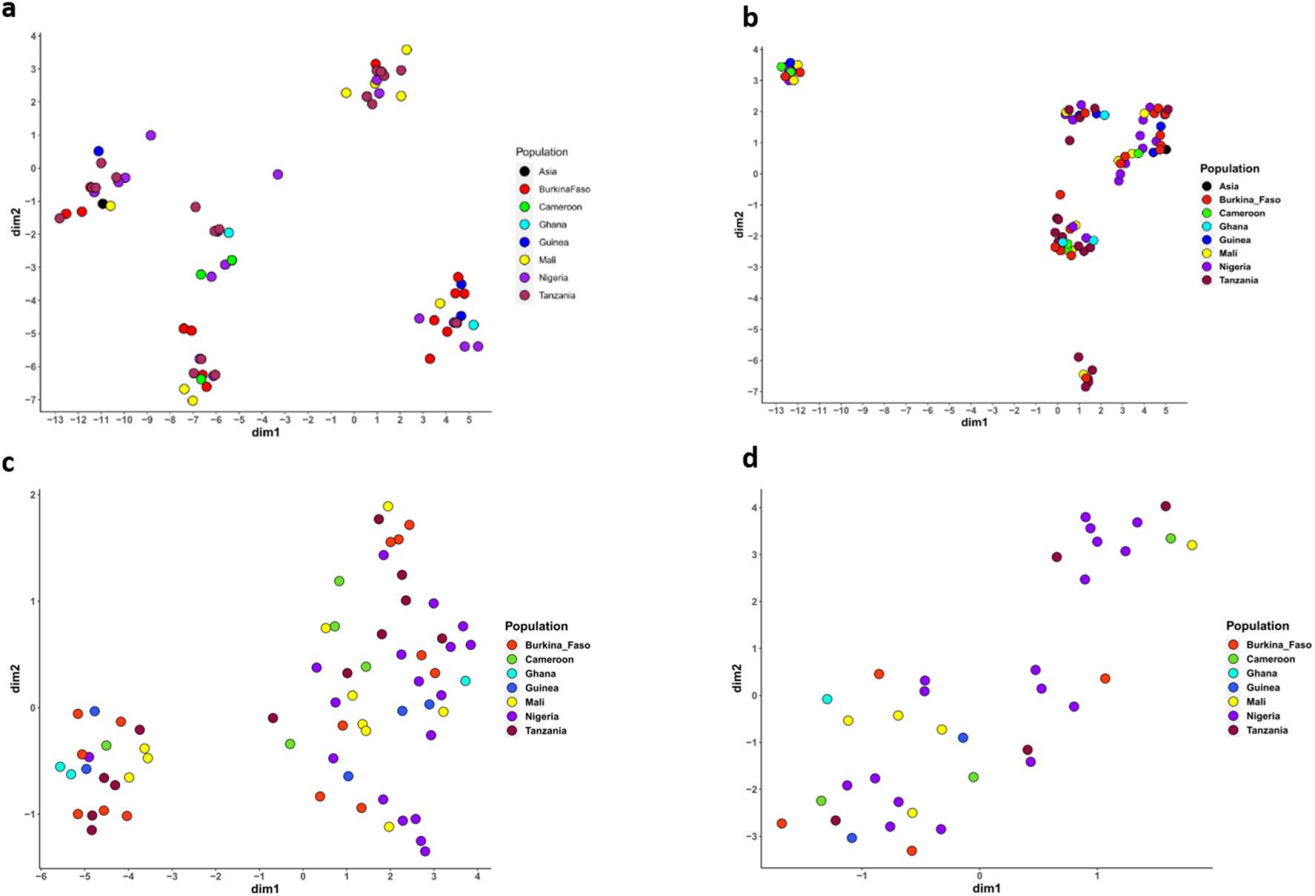
Discriminant analysis of principal component (DAPC) scatter display of population clusters of *P. malariae* isolates by (**a**) microsatellites, (**b**) SNP unfiltered, (**c**) SNP filtered for missingness only and (**d**) SNP denoised and filtered.

**Figure 4:**
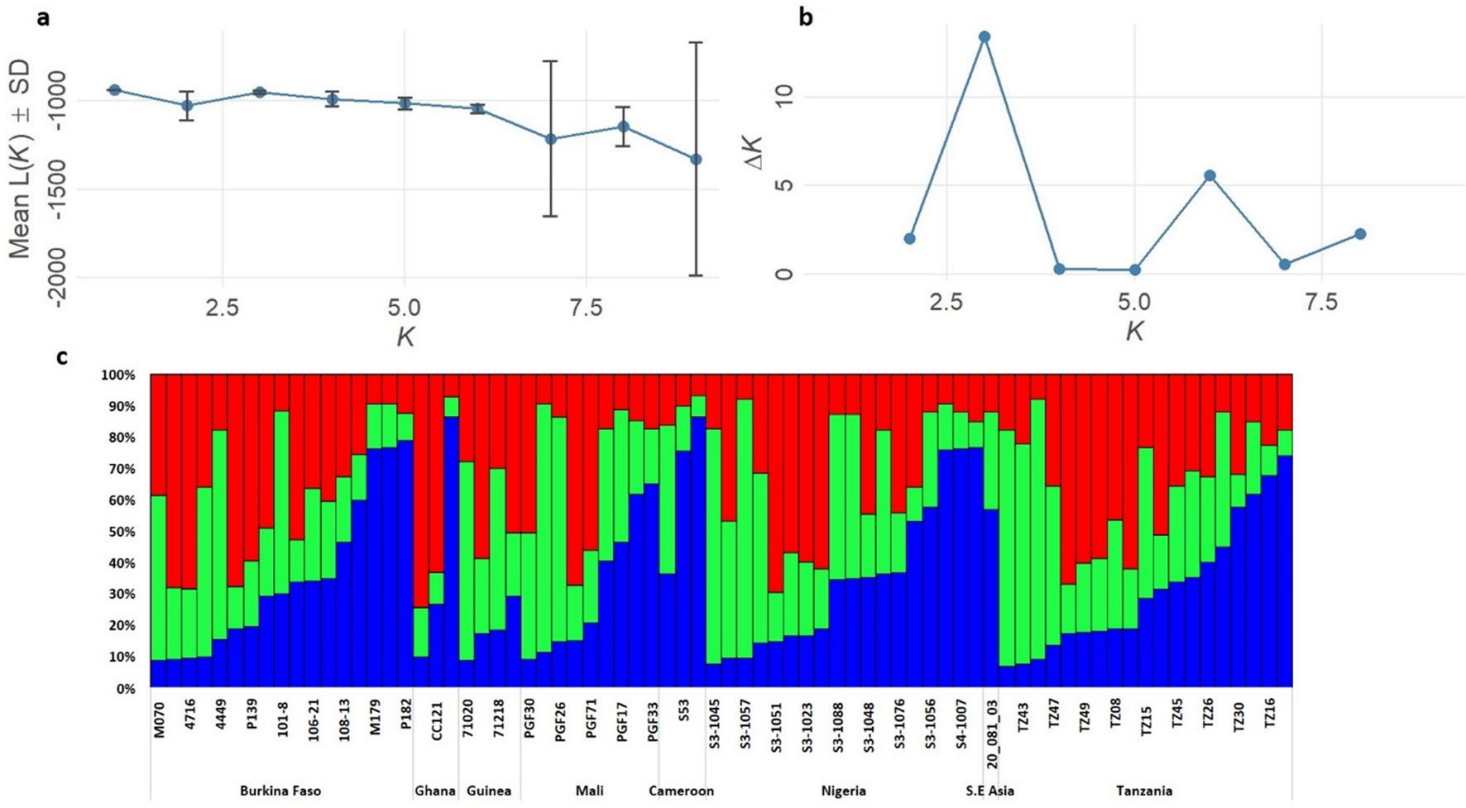
Population differentiation and ancestry using STRUCTURE showing optimal *K* value (*K* = 3) by the method of Evanno (**a**) mean likelihood of *K* and (**b**) *ΔK*. (**c**) Bar plot of individual Bayesian assignment probabilities of microsatellites for *P. malariae* from different countries with admixture model.

Further, microsatellite data identified five genetic clusters, which by DAPC scatter display were not also defined by the geographic origin of the isolates. Similarly, SNP based clustering using the unfiltered dataset grouped the isolates into five clusters with each cluster having isolates from different geographic origins (Fig. 3). While the pattern of geographic-independent clustering was still observed with the filtered and denoised datasets, the clusters were less defined from a reduced number of isolates especially in the denoised data (Fig. 3c,d). Using STRUCTURE software, the optimal number of clusters by Evanno method^19^ was defined as *K* = 3 (Fig. 4a,b), though additional peaks were detected in the Evanno graph at *K* = 6 and *K* = 8. Considering 70% as the probability threshold to assign an individual to a particular cluster, admixture modelling grouped isolates into three ancestral clusters (Fig. 4c), none of which was unique to isolates from any geographic setting.

**Figure 5:**
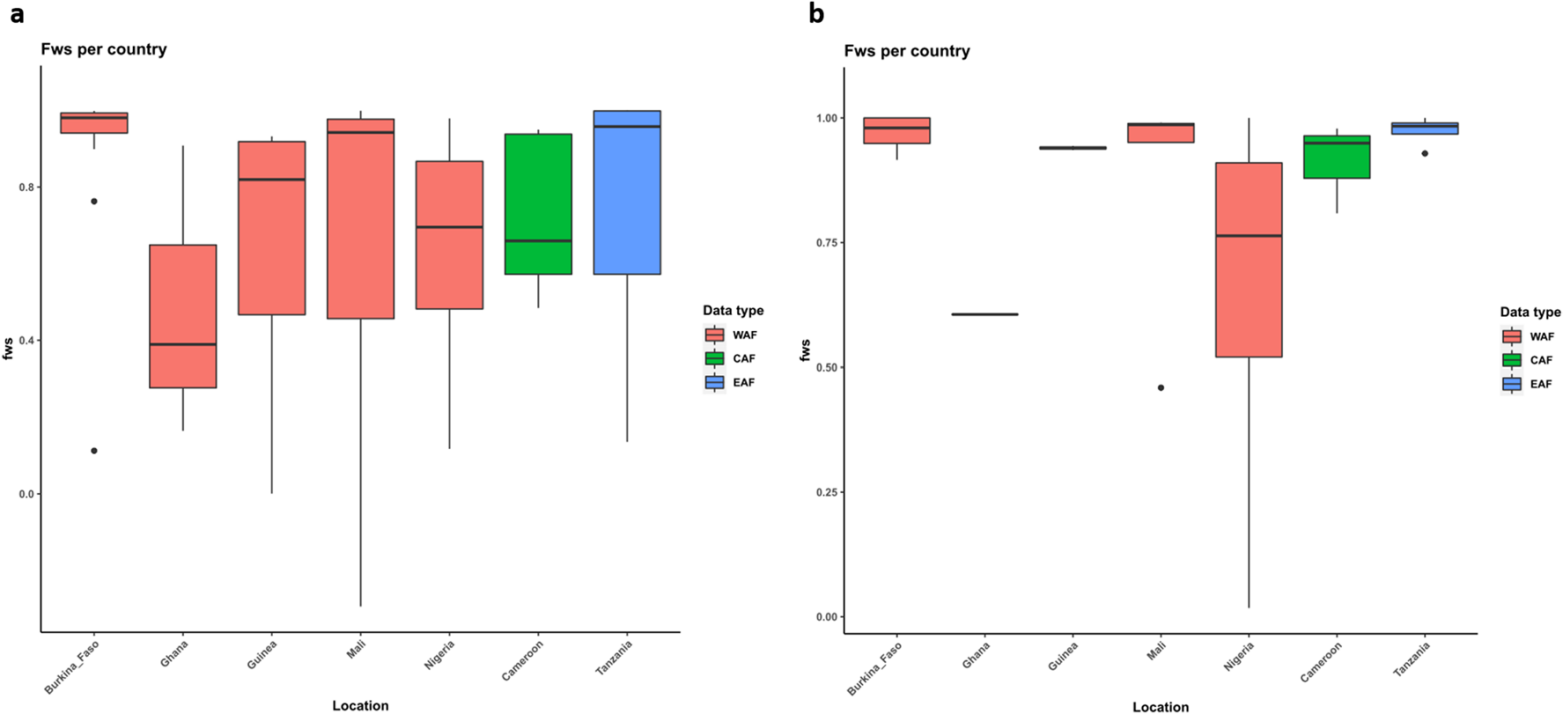
Complexity of infection across the different countries represented by Wright’s inbreeding co-efficient (*Fws*) using (**a**) SNP unfiltered data and (**b**) SNP denoised and filtered data.

Mixed genotyped infections (*F*_*WS*_ values < 0.95) were identified in the SNP data from all populations, though most isolates had high Fws from the denoised data, suggesting a single dominant genotype (Fig. 5a,b). Isolates from Nigeria and Ghana had the lowest mean *Fws*, thus more complex.

### SNPs at orthologous drug resistance loci

Sequence alignment of the orthologous drug-resistance genes with BOWTIE identified five more mutations from the unmerged reads compared to the merged reads with both variant calling tools, whereas the merged reads identified significantly more variants with BWA sequence alignment (Supplementary Tables 2 and 3). Combining all the sequence alignment and variant calling algorithms, twenty concordant SNPs from the two approaches (though occurring in low frequencies) were retained; of which seven were retained from the denoised and filtered dataset (Table 6). Eight of these coded for nonsynonymous variants, resulted in amino acid substitutions and interestingly, three of the four concordant SNPs reported in *Pmaat1* were nonsynonymous. The nonsynonymous SNP observed in *P. malariae* sulfadoxine resistance gene (*Pmdhps*) at position S452C aligned in close proximity with the S436A/F mutation in *P. falciparum*. This was also the case for the nonsynonymous SNP observed in the multi-drug resistance gene (*Pmmdr1*) at position V100L, lying in close proximity to the N86Y/F mutation in *P. falciparum*.

**Table 6:** Single nucleotide polymorphisms (SNPs) detected in orthologous *P. malariae* drug resistance genes.

S/N	Gene	pos	ref	alt	Mutation type	Amino acid codon change	Amino acid change	Effect	Frequency (%)	Retained after denoising
1	*Pmcrt*	348	C	G	Transversion	ACC/ACG	Thr/Thr	Synonymous	2.53	No
2	*Pmmdr1*	1389	C	T	Transition	AGC/AGT	Ser/Ser	Synonymous	10.13	Yes
3	*Pmmdr1*	1743	A	T	Transversion	GGA/GGT	Gly/Gly	Synonymous	1.27	No
4	*Pmmdr1*	1845	G	A	Transition	TTG/TTA	Leu/Leu	Synonymous	6.33	Yes
5	*Pmmdr1*	298	G	T	Transversion	GTA/TTA	Val/Leu	Non-synonymous	1.27	No
6	*Pmaat1*	1201	G	C	Transversion	GTT/CTT	Val/Leu	Non-synonymous	2.53	No
7	*Pmaat1*	183	C	T	Transition	AGC/AGT	Ser/Ser	Synonymous	3.79	No
8	*Pmaat1*	434	A	G	Transition	AAT/AGT	Asn/Ser	Non-synonymous	1.27	No
9	*Pmaat1*	451	T	A	Transversion	TTG/ATG	Leu/Met	Non-synonymous	1.27	No
10	*Pmatp4*	3129	A	G	Transition	TTA/TTG	Leu/Leu	Synonymous	2.53	No
11	*Pmatp4*	603	G	C	Transversion	GGG/GGC	Gly/Gly	Synonymous	2.53	No
12	*Pmnhe*	591	A	G	Transition	TCA/TCG	Ser/Ser	Synonymous	1.27	No
13	*Pmdhps*	1879	A	T	Transversion	AGC/TGC	Ser/Cys	Non-synonymous	1.27	No
14	*Pmcytb*	412	C	T	Transition	CTA/TTA	Leu/Leu	Synonymous	1.27	No
15	*Pmcytb*	479	G	A	Transition	GGT/GAT	Gly/Asp	Non-synonymous	1.27	No
16	*Pmcytb*	528	T	C	Transition	TAT/TAC	Tyr/Tyr	Synonymous	3.79	Yes
17	*Pmcytb*	668	A	G	Transition	AAT/AGT	Asn/Ser	Non-synonymous	1.27	Yes
18	*Pmcytb*	690	T	C,A	Transition /Transversion	TTT/TTC,TTA	Phe/Phe,Leu	Synonymous, Non-synonymous	7.59	Yes
19	*Pmcytb*	708	A	T	Transversion	GCA/GCT	Ala/Ala	Synonymous	3.79	Yes
20	*Pmcytb*	819	T	A	Transversion	ATT/ATA	Ile/Ile	Synonymous	2.53	Yes

**Figure 6:**
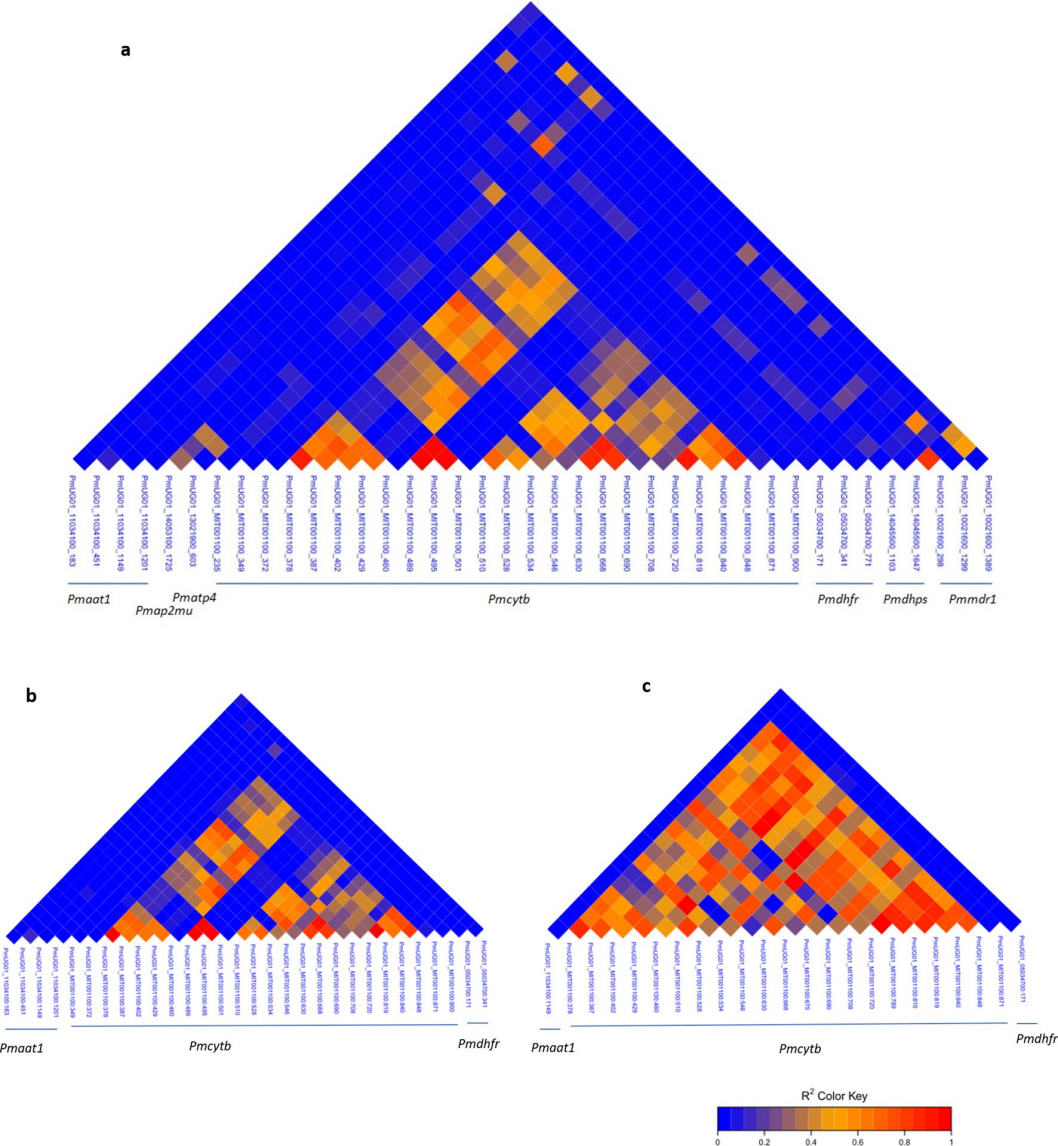
Linkage disequilibrium between SNPs of orthologous drug resistance genes computed from (**a**) unfiltered data, (**b**) data filtered for missingness and (**c**) denoised and filtered data.

The linkage disequilibrium (LD) heat map revealed significant patterns of LD between SNPs on different orthologous drug resistance genes. High r^2^ values were observed between most of the SNPs on the mitochondrial *Pmcytb* gene and between *Pmcytb* SNPs and those on other targets such as *Pmdhps, Pmdhfr* and *Pmmdr1* from the unfiltered dataset (Fig. 6a). The high LD observed in the mitochondrial SNPs were retained in both the filtered and denoised datasets (Fig. 6b,c).

## Discussion and conclusion

Malaria elimination programs and tools are focused on eliminating the main malaria parasites *P. falciparum* and *P. vivax*, but other malaria parasites species such as *P. malariae* are co-transmitted in malaria endemic regions and warrant attention for achieving elimination. As knowledge of the diversity and effect of elimination tools on these minor species have not had much academic or public health focus, this study took advantage of the Pathogen Diversity Network Africa (PDNA), to collate a small but the widest sample set of *P. malariae* isolates across seven African countries and one from Asia, describing population structure, high genetic diversity, genotypic richness and evenness in the species of malaria parasite.

A high level of genetic diversity is essential for the long-term survival of populations, and the extent of variation determines the ability of the species to adapt to environmental challenges imposed by nature or control interventions. The high diversity in *P. malariae* found here is similar to those previously reported in Kenya and Malawi^2,3^, despite the variable and small number of samples analysed from some of the countries. Malaria transmission intensity and history of interventions vary across the different countries represented in this study and could have affected the results. High transmission results in frequent heterologous recombination of the parasite in the mosquito vector, breaking down linkage disequilibrium between variable loci and increasing the genetic diversity within populations. In general, the probability of outcrossing in parasite populations vary from a high to low malaria transmission gradient in the West, Central and Eastly direction in sub-Saharan Africa^20^. This notwithstanding, heterozygosity was high and genetic distance was low between isolates despite the differences in malaria transmission intensity, except for the three isolates from Cameroon. The low genetic distance observed in this study require further validation since there are no studies on *P. malariae* for direct comparison. However, the results obtained are consistent with a recent *P. falciparum* study in Nigeria^21^ howbeit in sharp contrast with an older study in Senegal^22^, suggesting relatively high levels of panmixia in the current sampled populations despite the varying transmission patterns. Indeed, multi-locus genotypes were rare across all populations, an indication of recombination and absence of the clonal expansion observed in some *P. falciparum* populations with low or seasonal malaria transmission^23^.

Recurrent gene flow promotion between parasite populations across countries via human or vector migration, could have led to a lack of differentiation according to geographic origin^3^. This was evident in low microsatellite differentiation indices between countries, although the small number of isolates per country population limits the accuracy of the indices inferred. It is also possible that gene flow alone does not explain the high genetic diversity or lack of geographic differentiation observed in *P. malariae*, other factors such as a lack of bottleneck event or intervention to reduce local diversity could also be considered.

Population structure analysis with neutral microsatellite loci (i.e SSRs) identified five clusters, each with isolates from different countries. This is further indication of high intrapopulation variability in these markers and absence of population specific selection of these loci that may drive population differentiation. This structure is not consistent with isolation by distance seen for *P. falciparum*, where genetic clusters can be allocated to geographic populations in the west, central and eastern African regions. As *P. malariae* mostly occurs as coinfections with *P. falciparum*, the drivers of such independent substructure may therefore be different, or it is possible that this could have been established by an earlier event preceding any population drift due to demography or isolation. Using the admixture model in STRUCTURE, an optimum of three ancestral clusters were determined and this also showed all isolates with components of each ancestry irrespective of the country of origin. STRUCTURE implements a Bayesian algorithm to identify groups of individuals at Hardy–Weinberg and linkage equilibrium. However, its robustness was shown to be impacted by small uneven sample sizes between subpopulations and/or hierarchical levels of population structure^24,25^.

The SNP data from selected *P. malariae* orthologues of *P. falciparum* drug resistance genes also clustered isolates into 5 subpopulations, although only 3 less distinct clusters were retained after stringent filtration processes, and membership of the subgroups did not overlap fully with those determined by microsatellites. The distribution between the SNP and microsatellite distance differed, with wider distances between smaller number of isolates using SSR data. This is expected as SSRs are multi-allelic, likely neutral, and more likely to differ between pairs of isolates. Thus, further investigation into the possible drivers of population differentiation for this parasite species will improve understanding of its complexity, particularly with regard to control and elimination strategies. While it appeared that most infections had mixed genomes (polygenomic) as indicated by *Fws*, this was affected by the denoising and filtration pipelines used for analysis. Thus, further investigation using appropriate sample size will be necessary to clarify if co-transmission of different clones and increased possibility of recombination exists for this Plasmodium species. High level of infection complexity is important to note, given that it is one of the indices for monitoring the effect of interventions. Unlike for *P. falciparum*, the complexity did not seem to be higher in relatively higher malaria transmission settings and may be part of the unique biology of this species that needs further investigation. As drugs and other interventions drive down populations, selection and changes in complexity should be monitored for the species.

Drugs have been a major selective force on *P. falciparum*, with resistance associated with mutations in several genes and signatures of positive selection across the genomes. We identified 20 *P. malariae* mutations in the orthologous drug resistant genes by combining different sequence alignment and variant calling algorithms. These putative variants have not been described in previous targeted or genome scans, probably because of differences in isolates used or the methods applied. Here we retained only high-quality variants supported by combinations of two mapping and two SNP calling algorithms. Most of the candidate variants were synonymous but there were several nonsynonymous SNPs across seven genes, especially in *Pmcytb*, whose *P. falciparum* orthologue drive resistance against atovaquone. Atovaquone is a member of the quinolines, to which resistance in *P. falciparum* have been associated with mutations in multidrug resistance gene (*Pfmdr1*), chloroquine resistance transporter (*Pfcrt*) and an amino acid transporter (*Pfaat1*). Most of the LD observed with the unfiltered dataset were not reproducible with the denoised and filtered dataset, with the exception of the LD observed in the SNPs of the mitochondrial gene, either due to common ancestry or selection of dominant haplotype by drugs or other factors. Additional candidate variants were seen in *Pmdhfr* and *Pmdhps,* orthologues of antifolate resistance in *P. falciparum*. The antifolate antimalarials, sulfadoxine-pyrimethamine are still in wide use for chemoprevention against malaria in pregnancy and combination with amodiaquine for seasonal malaria chemoprevention in West Africa. These together could be selecting for the identified variants. While the nonsynonymous SNPs reported here occurred in low frequencies, further verification, characterization and association of these SNPs will require increased genomic surveillance and phenotype association studies from in vivo and ex vivo therapeutic efficacy tests.

A limitation of this study, which warrants cautious interpretation of the results, is the small number of samples analyzed across the different countries and the lack of bio and clinical data of the samples. Larger population studies for *P. malariae* with appropriate epidemiology or clinical data are required to validate the findings of smaller studies as reported here. Another limitation is the use of standard bioinformatics pipelines designed for *P. falciparum*. While these may be acceptable for preliminary analysis, custom pipelines that take possible amplification and sequencing errors into consideration may be better for *P. malariae*, particularly because of the scarcity of population data with high quality confirmed variants from this parasite species. The different sample types analyzed could also be a limitation to this study, the dried blood spot samples were more amenable to producing poor quality results, possibly due to the low prevalence and low parasite density of the non-falciparum species. Therefore, venous blood sampling and more robust molecular techniques that take these into consideration will be beneficial in future molecular surveillance of *P. malariae*.

The current drive for malaria elimination needs innovative strategies to target all malaria parasites. One approach can be by integrating genomic surveillance of all *Plasmodium* species into malaria control and elimination programs in sub-Saharan Africa, learning from the experience with COVID-19, to refine approaches as new variants are identified and monitored. This study has established the relevance of this in *P. malariae*.

## Supplementary Information

Supplementary Information.

## Data Availability

The data for this study have been deposited in the European Nucleotide Archive (ENA) at EMBL-EBI under accession number PRJEB55468 (https://www.ebi.ac.uk/ena/browser/view/PRJEB55468). Pipeline for data analysis and scripts are available on Github https://github.com/MPB-mrcg/P.Malaria_antimalaria_resistance_genes.git.
