# Supplementary material for: Plasmodium malariae structure and genetic diversity in sub-Saharan Africa determined from microsatellite variants and linked SNPs in orthologues of antimalarial resistance genes
Source: Sci Rep. 2022 Dec 19;12:21881. doi: 10.1038/s41598-022-26625-w (PMC9761029; doi:10.1038/s41598-022-26625-w)
Supplement: Supplementary file 1 — Supplementary Information. [file 41598_2022_26625_MOESM1_ESM.docx]

**Supplementary document**

*Plasmodium malariae* sub-structure in sub-Saharan Africa determined from microsatellite variants and linked SNPs in orthologues of antimalarial resistance genes.


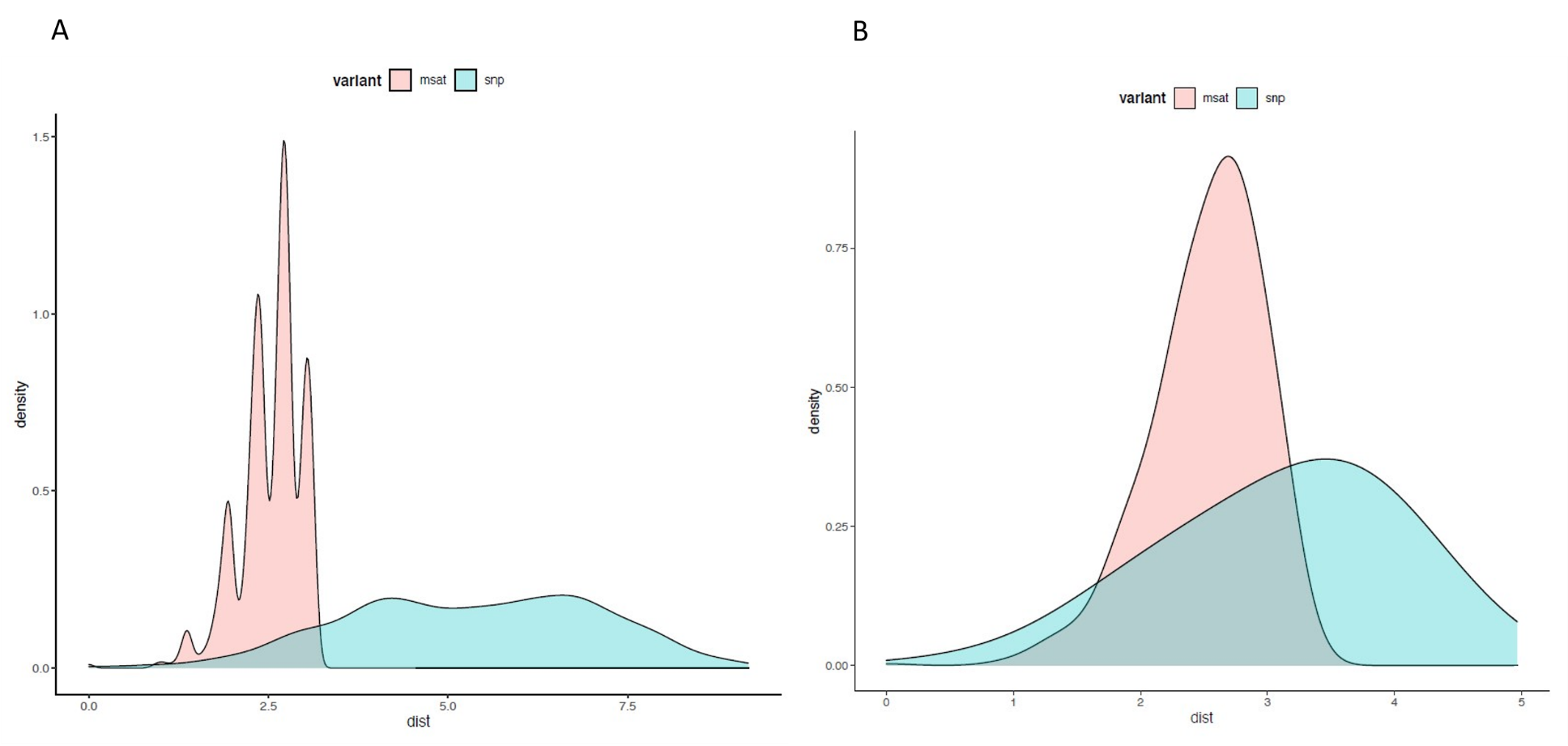


Supplementary Figure 1: Distribution plot of genetic distances (A) unfiltered SNP data (B) filtered SNP data


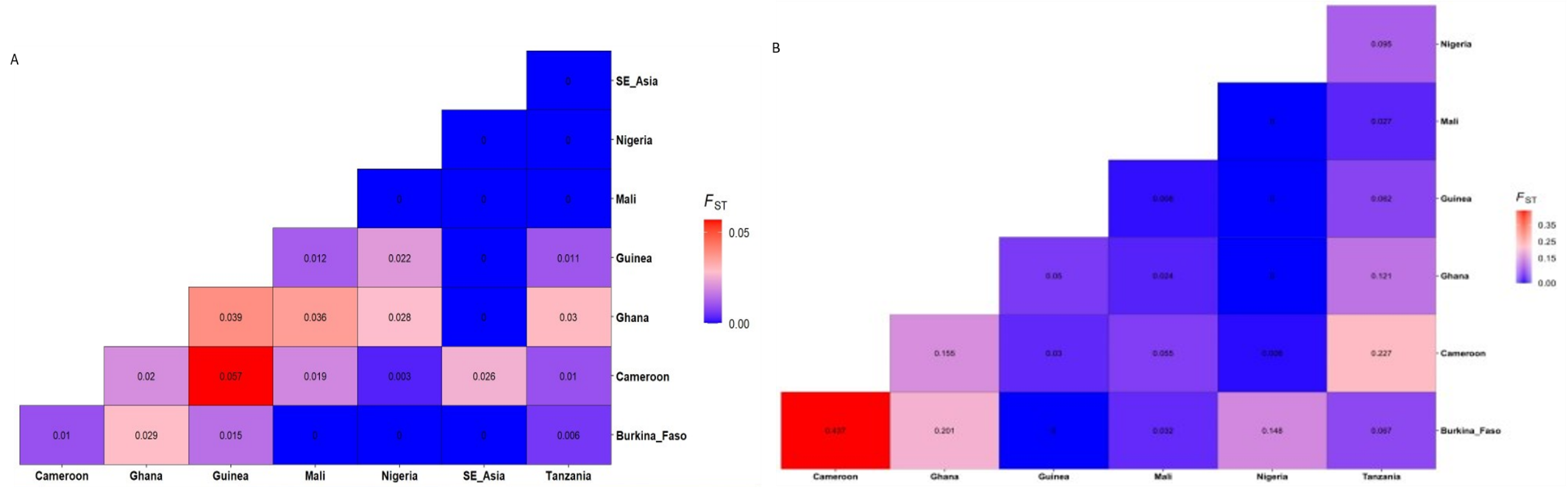


Supplementary Figure 2: Pairwise F_ST_ map (*pairwisefst* command in R Version 4.1.13) showing levels of genetic differentiation between populations with (A) microsatellite data (B) SNP data

Supplementary Table 1: Summary of data before and after filtration

| Data type | # variants before filtration | # variants after filtration | # samples before filtration | # samples after filtration |
| --- | --- | --- | --- | --- |
| Mitochondrial data | 25 | 23 | 77 | 67 |
| Mitochondrial data (denoised) | 20 | 20 | 73 | 35 |
| Non-mitochondrial data | 19 | 10 | 77 | 42 |
| Non-mitochondrial data (denoised) | 7 | 5 | 73 | 19 |
| All data | 44 | 34 | 77 | 67 |
| All data (denoised) | 27 | 25 | 73 | 36 |

Supplementary Table 2: Variants called by BOWTIE alignment using either merged or unmerged reads.

| Bowtie_merged | | | | | | Bowtie_unmerged | | | | | |
| --- | --- | --- | --- | --- | --- | --- | --- | --- | --- | --- | --- |
| gene_ID | gene | pos | ref | bcf_alt | FBayes_alt | gene |  | pos | ref | bcf_alt | FBayes_alt |
| PmCRT_amplicon | PmCRT | 348 | C | G | G | PmCRT_amplicon | PmCRT | 348 | C | G | G |
| PmUG01_05034700 | PmDHFR | 341 | A | G | G | PmUG01_05034700 | PmDHFR | 341 | A | G | G |
| PmUG01_10021600 | PmMDR1 | 1299 | C | T | T | PmUG01_05034700 | PmDHFR | 771 | C | G | G |
| PmUG01_10021600 | PmMDR1 | 1389 | C | T | T | PmUG01_10021600 | PmMDR1 | 1389 | C | T | T |
| PmUG01_10021600 | PmMDR1 | 1743 | A | T | T | PmUG01_10021600 | PmMDR1 | 1743 | A | T | T |
| PmUG01_10021600 | PmMDR1 | 1845 | G | A | A | PmUG01_10021600 | PmMDR1 | 1845 | G | A | A |
| PmUG01_10021600 | PmMDR1 | 298 | G | T | T | PmUG01_10021600 | PmMDR1 | 298 | G | T | T |
| PmUG01_11034100 | PmAAT1 | 1201 | G | C | C | PmUG01_11034100 | PmAAT1 | 1149 | G | T | T |
| PmUG01_11034100 | PmAAT1 | 183 | C | T | T | PmUG01_11034100 | PmAAT1 | 1201 | G | C | C |
| PmUG01_11034100 | PmAAT1 | 434 | A | G | G | PmUG01_11034100 | PmAAT1 | 183 | C | T | T |
| PmUG01_11034100 | PmAAT1 | 451 | T | A | A | PmUG01_11034100 | PmAAT1 | 434 | A | G | G |
| PmUG01_13021900 | PmATP4 | 3129 | A | G | G | PmUG01_11034100 | PmAAT1 | 451 | T | A | A |
| PmUG01_13021900 | PmATP4 | 603 | G | C | C | PmUG01_13021900 | PmATP4 | 3129 | A | G | G |
| PmUG01_14020100 | PmNHE | 3926 | A | T | T | PmUG01_13021900 | PmATP4 | 603 | G | C | C |
| PmUG01_14020100 | PmNHE | 591 | A | G | G | PmUG01_14020100 | PmNHE | 591 | A | G | G |
| PmUG01_14045500 | PmDHPS | 1614 | G | A | A | PmUG01_14045500 | PmDHPS | 1103 | G | T | T |
| PmUG01_14045500 | PmDHPS | 1879 | A | T | T | PmUG01_14045500 | PmDHPS | 1879 | A | T | T |
| PmUG01_MIT001100 | PmCYTB | 412 | C | T | T | PmUG01_MIT001100 | PmCYTB | 402 | T | C | C |
| PmUG01_MIT001100 | PmCYTB | 479 | G | A | A | PmUG01_MIT001100 | PmCYTB | 412 | C | T | T |
| PmUG01_MIT001100 | PmCYTB | 516 | T | G | G | PmUG01_MIT001100 | PmCYTB | 429 | A | T | T |
| PmUG01_MIT001100 | PmCYTB | 528 | T | C | C | PmUG01_MIT001100 | PmCYTB | 463 | C | T | T |
| PmUG01_MIT001100 | PmCYTB | 540 | A | T | T | PmUG01_MIT001100 | PmCYTB | 479 | G | A | A |
| PmUG01_MIT001100 | PmCYTB | 668 | A | G | G | PmUG01_MIT001100 | PmCYTB | 516 | T | G | G |
| PmUG01_MIT001100 | PmCYTB | 690 | T | C,A | C,A | PmUG01_MIT001100 | PmCYTB | 524 | C | A | A |
| PmUG01_MIT001100 | PmCYTB | 708 | A | T | T | PmUG01_MIT001100 | PmCYTB | 528 | T | C | C |
| PmUG01_MIT001100 | PmCYTB | 819 | T | A | A | PmUG01_MIT001100 | PmCYTB | 540 | A | T | T |
| PmUG01_MIT001100 | PmCYTB | 840 | T | G | G | PmUG01_MIT001100 | PmCYTB | 630 | A | G | G |
| PmUG01_MIT001100 | PmCYTB | 848 | C | T | T | PmUG01_MIT001100 | PmCYTB | 668 | A | G | G |
| PmUG01_MIT001100 | PmCYTB | 885 | C | T | A | PmUG01_MIT001100 | PmCYTB | 675 | T | C | C |
|  |  |  |  |  |  | PmUG01_MIT001100 | PmCYTB | 690 | T | C,A | A,C |
|  |  |  |  |  |  | PmUG01_MIT001100 | PmCYTB | 708 | A | T | T |
|  |  |  |  |  |  | PmUG01_MIT001100 | PmCYTB | 789 | A | T | T |
|  |  |  |  |  |  | PmUG01_MIT001100 | PmCYTB | 811 | C | T | T |
|  |  |  |  |  |  | PmUG01_MIT001100 | PmCYTB | 819 | T | A | A |

Supplementary Table 3: Variants called by BWA alignment using either merged or unmerged reads.

| bwa_merged | | | | | | bwa_unmerged | | | | | |
| --- | --- | --- | --- | --- | --- | --- | --- | --- | --- | --- | --- |
| gene |  | pos | ref | bcf_alt | FBayes_alt | gene |  | pos | ref | bcf_alt | FBayes_alt |
| PmCRT_amplicon | PmCRT | 228 | C | G | T | PmCRT_amplicon | PmCRT | 348 | C | G | G |
| PmCRT_amplicon | PmCRT | 348 | C | G | G | PmUG01_05034700 | PmDHFR | 341 | A | G | G |
| PmUG01_02017400 | PmATP6 | 3945 | T | A | A | PmUG01_05034700 | PmDHFR | 771 | C | G | G |
| PmUG01_05034700 | PmDHFR | 771 | C | G | G | PmUG01_10021600 | PmMDR1 | 1299 | C | T | T |
| PmUG01_10021600 | PmMDR1 | 1299 | C | T | T | PmUG01_10021600 | PmMDR1 | 1389 | C | T | T |
| PmUG01_10021600 | PmMDR1 | 1389 | C | T | T | PmUG01_10021600 | PmMDR1 | 1743 | A | T | T |
| PmUG01_10021600 | PmMDR1 | 1743 | A | T | T | PmUG01_10021600 | PmMDR1 | 1845 | G | A | A |
| PmUG01_10021600 | PmMDR1 | 1845 | G | A | A | PmUG01_10021600 | PmMDR1 | 298 | G | T | T |
| PmUG01_10021600 | PmMDR1 | 298 | G | T | T | PmUG01_11034100 | PmAAT1 | 1149 | G | T | T |
| PmUG01_10021600 | PmMDR1 | 4002 | G | A | A | PmUG01_11034100 | PmAAT1 | 1201 | G | C | C |
| PmUG01_10021600 | PmMDR1 | 4008 | T | A | A | PmUG01_11034100 | PmAAT1 | 183 | C | T | T |
| PmUG01_10021600 | PmMDR1 | 900 | G | T | T | PmUG01_11034100 | PmAAT1 | 434 | A | G | G |
| PmUG01_11034100 | PmAAT1 | 1149 | G | T | T | PmUG01_11034100 | PmAAT1 | 451 | T | A | A |
| PmUG01_11034100 | PmAAT1 | 1201 | G | C | C | PmUG01_13021900 | PmATP4 | 3129 | A | G | G |
| PmUG01_11034100 | PmAAT1 | 183 | C | T | T | PmUG01_13021900 | PmATP4 | 603 | G | C | C |
| PmUG01_11034100 | PmAAT1 | 434 | A | G | G | PmUG01_14020100 | PmNHE | 3926 | A | T | T |
| PmUG01_11034100 | PmAAT1 | 451 | T | A | A | PmUG01_14020100 | PmNHE | 591 | A | G | G |
| PmUG01_12021200 | PmKelch13 | 1964 | C | A | A | PmUG01_14020100 | PmNHE | 765 | G | A | A |
| PmUG01_12021200 | PmKelch13 | 351 | T | G | G | PmUG01_14020100 | PmNHE | 780 | A | T | T |
| PmUG01_13021900 | ATP4 | 1002 | GGTAGTAGTAG | GGTAGTAG | GGTAGTAG | PmUG01_14020100 | PmNHE | 861 | C | T | T |
| PmUG01_13021900 | ATP4 | 1874 | T | A | A | PmUG01_14045500 | PmDHPS | 1103 | G | T | T |
| PmUG01_13021900 | ATP4 | 2184 | T | G | G | PmUG01_14045500 | PmDHPS | 1614 | G | A | A |
| PmUG01_13021900 | ATP4 | 242 | C | A | A | PmUG01_14045500 | PmDHPS | 1879 | A | T | T |
| PmUG01_13021900 | ATP4 | 3129 | A | G | G | PmUG01_MIT001100 | PmCYTB | 153 | T | C | C |
| PmUG01_13021900 | ATP4 | 603 | G | C | C | PmUG01_MIT001100 | PmCYTB | 165 | A | T | T |
| PmUG01_14020100 | PmNHE | 2877 | C | T | T | PmUG01_MIT001100 | PmCYTB | 235 | T | A | A |
| PmUG01_14020100 | PmNHE | 3926 | A | T | T | PmUG01_MIT001100 | PmCYTB | 255 | A | G | G |
| PmUG01_14020100 | PmNHE | 4461 | C | A | A | PmUG01_MIT001100 | PmCYTB | 274 | A | C | C |
| PmUG01_14020100 | PmNHE | 591 | A | G | G | PmUG01_MIT001100 | PmCYTB | 286 | T | C | C |
| PmUG01_14020100 | PmNHE | 765 | G | A | A | PmUG01_MIT001100 | PmCYTB | 378 | C | T | T |
| PmUG01_14020100 | PmNHE | 780 | A | T | T | PmUG01_MIT001100 | PmCYTB | 387 | T | A | A |
| PmUG01_14020100 | PmNHE | 852 | T | A | A | PmUG01_MIT001100 | PmCYTB | 402 | T | C | C |
| PmUG01_14020100 | PmNHE | 861 | C | T | T | PmUG01_MIT001100 | PmCYTB | 412 | C | T | T |
| PmUG01_14020100 | PmNHE | 876 | T | A | A | PmUG01_MIT001100 | PmCYTB | 429 | A | T | T |
| PmUG01_14045500 | PmDHPS | 1220 | A | T,G | G,T | PmUG01_MIT001100 | PmCYTB | 479 | G | A | A |
| PmUG01_14045500 | PmDHPS | 1879 | A | T | T | PmUG01_MIT001100 | PmCYTB | 524 | C | A | A |
| PmUG01_14045500 | PmDHPS | 523 | C | A | A | PmUG01_MIT001100 | PmCYTB | 528 | T | C | C |
| PmUG01_14045500 | PmDHPS | 657 | C | A | A | PmUG01_MIT001100 | PmCYTB | 546 | T | A | A |
| PmUG01_14053100 | PmAP2mu | 1452 | A | G | G | PmUG01_MIT001100 | PmCYTB | 582 | G | A | A |
| PmUG01_MIT001100 | PmCYTB | 102 | A | T | T | PmUG01_MIT001100 | PmCYTB | 630 | A | G | G |
| PmUG01_MIT001100 | PmCYTB | 153 | T | C | C | PmUG01_MIT001100 | PmCYTB | 668 | A | G | G |
| PmUG01_MIT001100 | PmCYTB | 165 | A | T | T | PmUG01_MIT001100 | PmCYTB | 675 | T | C | C,A |
| PmUG01_MIT001100 | PmCYTB | 235 | T | A | A | PmUG01_MIT001100 | PmCYTB | 690 | T | C,A | C,A |
| PmUG01_MIT001100 | PmCYTB | 255 | A | G | G | PmUG01_MIT001100 | PmCYTB | 708 | A | T | T |
| PmUG01_MIT001100 | PmCYTB | 274 | A | C | C | PmUG01_MIT001100 | PmCYTB | 720 | T | A | A |
| PmUG01_MIT001100 | PmCYTB | 286 | T | C | C | PmUG01_MIT001100 | PmCYTB | 819 | T | A | A |
| PmUG01_MIT001100 | PmCYTB | 375 | G | C | C | PmUG01_MIT001100 | PmCYTB | 840 | T | G | G |
| PmUG01_MIT001100 | PmCYTB | 378 | C | T | T | PmUG01_MIT001100 | PmCYTB | 848 | C | T | T |
| PmUG01_MIT001100 | PmCYTB | 387 | T | A | A | PmUG01_MIT001100 | PmCYTB | 871 | T | C | C |
| PmUG01_MIT001100 | PmCYTB | 402 | T | C | C | PmUG01_MIT001100 | PmCYTB | 885 | C | T | A,T |
| PmUG01_MIT001100 | PmCYTB | 412 | C | T | T |  |  |  |  |  |  |
| PmUG01_MIT001100 | PmCYTB | 429 | A | T | T |  |  |  |  |  |  |
| PmUG01_MIT001100 | PmCYTB | 479 | G | A | A |  |  |  |  |  |  |
| PmUG01_MIT001100 | PmCYTB | 528 | T | C | C |  |  |  |  |  |  |
| PmUG01_MIT001100 | PmCYTB | 546 | T | A | A |  |  |  |  |  |  |
| PmUG01_MIT001100 | PmCYTB | 582 | G | A | A |  |  |  |  |  |  |
| PmUG01_MIT001100 | PmCYTB | 6 | T | C | C |  |  |  |  |  |  |
| PmUG01_MIT001100 | PmCYTB | 630 | A | G | G |  |  |  |  |  |  |
| PmUG01_MIT001100 | PmCYTB | 668 | A | G | G |  |  |  |  |  |  |
| PmUG01_MIT001100 | PmCYTB | 675 | T | C | C,A |  |  |  |  |  |  |
| PmUG01_MIT001100 | PmCYTB | 690 | T | C,A | C,A |  |  |  |  |  |  |
| PmUG01_MIT001100 | PmCYTB | 708 | A | T | T |  |  |  |  |  |  |
| PmUG01_MIT001100 | PmCYTB | 720 | T | A | A |  |  |  |  |  |  |
| PmUG01_MIT001100 | PmCYTB | 819 | T | A | A |  |  |  |  |  |  |
| PmUG01_MIT001100 | PmCYTB | 840 | T | G | G |  |  |  |  |  |  |
| PmUG01_MIT001100 | PmCYTB | 848 | C | T | T |  |  |  |  |  |  |
| PmUG01_MIT001100 | PmCYTB | 885 | C | T | A,T |  |  |  |  |  |  |
